# A neuronal correlate of insect stereopsis

**DOI:** 10.1038/s41467-019-10721-z

**Published:** 2019-06-28

**Authors:** Ronny Rosner, Joss von Hadeln, Ghaith Tarawneh, Jenny C. A. Read

**Affiliations:** 1 Institute of Neuroscience, Henry Wellcome Building for Neuroecology, Newcastle University, Framlington Place, Newcastle Upon Tyne, NE2 4HH UK; 2 Department of Biology, Animal Physiology & Center for Mind, Brain and Behavior, Philipps-Universität Marburg, 35032 Marburg, Germany

**Keywords:** Neural encoding, Oculomotor system, Object vision, Animal physiology, Entomology

## Abstract

A puzzle for neuroscience—and robotics—is how insects achieve surprisingly complex behaviours with such tiny brains. One example is depth perception via binocular stereopsis in the praying mantis, a predatory insect. Praying mantids use stereopsis, the computation of distances from disparities between the two retinal images, to trigger a raptorial strike of their forelegs when prey is within reach. The neuronal basis of this ability is entirely unknown. Here we show the first evidence that individual neurons in the praying mantis brain are tuned to specific disparities and eccentricities, and thus locations in 3D-space. Like disparity-tuned cortical cells in vertebrates, the responses of these mantis neurons are consistent with linear summation of binocular inputs followed by an output nonlinearity. Our study not only proves the existence of disparity sensitive neurons in an insect brain, it also reveals feedback connections hitherto undiscovered in any animal species.

## Abstract (web summary)

The praying mantis, a predatory insect, estimates depth via binocular vision. In this way, the animal decides whether prey is within reach. Here, the authors explore the neural correlates of binocular distance estimation and report that individual neurons are tuned to specific locations in 3D space.

## Introduction

In humans, stereopsis supports a rich perception of depth across the visual scene. This requires a complex network spanning multiple cortical areas and involving tens of millions of neurons^1,2^. Praying mantids achieve a form of stereopsis with a brain some five orders of magnitude smaller. Their stereopsis may do no more than estimate the probability that an already-fixated prey item is within catch range. Thus, it is natural to assume that insect stereopsis must be computed in a profoundly different and much simpler manner^3^. Insect stereopsis does differ from humans’ in using changes in luminance, rather than luminance directly^4^. However, this does not explain how the mantis brain combines information about the location of luminance changes in the two eyes. In primates, information from the two eyes is combined in individual neurons in the primary visual cortex, which are tuned to different retinal disparities (horizontal shifts of corresponding image features seen by both eyes) and thus different locations in 3D space. Such local computations are often regarded as far too elaborate and neuronally expensive for insect stereopsis^3,5,6^.

**Fig. 1:**
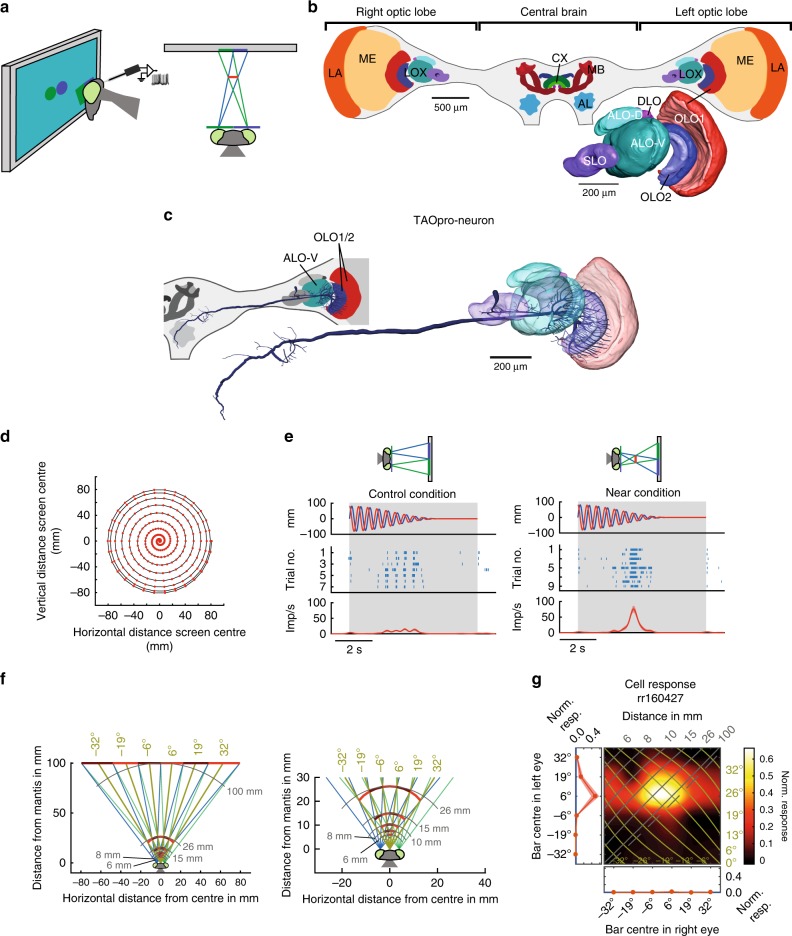
Experimental setup and disparity-sensitive TAOpro-neuron. **a** Praying mantis watches computer screen showing disc stimulus during neuronal recording (side view, top view). Spectral filters ensure each eye sees only one disc; lines of sight in blue (green) for left (right) eye. Virtual disc floats in front of screen (red line). **b** Mantis brain with major neuropils. Inset 3D-reconstruction of lobula complex (LOX), site of ramifications of all except one neuron type presented in this study. AL antennal lobe, ALO-D dorsal unit of the anterior lobe, ALO-V ventral unit of anterior lobe, CX central complex, LA lamina, MB mushroom bodies, ME medulla, OLO1/2 outer lobe 1/2, SLO stalk lobe. **c** Reconstruction of TAOpro-neuron (tangential projection neuron of the anterior and outer lobes) showing ramifications in LOX and central brain. Inset: left brain, colour shows LOX sub-compartments containing ramifications. **d** Disc trajectory. Red dots: disc centre at 60 Hz refresh rate. **e** TAOpro-neuron responses to disc stimulus. Sketches indicate stimulus disparity. Upper lanes: vertical (blue) and horizontal (red) distance of disc from screen centre; negative values are left and lower side of screen. Middle lanes raster plots, lower lanes spiking rates (average red line, ±1SEM) after Gaussian smoothing (SD 150 ms). Right plot: virtual disc at 25 mm (in catch range), left: control (right and left eye disc swapped). **f** Bar stimulus configuration with all 6 different bar positions on computer screen in 100 mm distance to animal (shown alternating dark/bright red for clarity; right panel zoom) and virtual bars (see also Supplementary Fig. 1 for further explanation). Azimuthal direction of simulated bars from mantis head midline in olive and distance isolines in grey. **g** Monocular and binocular responses to bar stimulus. Binocular responses as pseudocolour 2D-plot and monocular responses (averages ±1SEM; blue line background activity) as 1D-plots at left and bottom margins for respective eye. Axes show centre of bar shown to left and right eye, respectively. Binocular response is interpolated (raw plot in Supplementary Fig. 2). Isolines indicate azimuth (olive) and distance (grey) from mantis as shown in **f**. Dashed line marks screen locations implying objects at “infinity” (parallel lines of sight)

To determine whether neurons tuned to binocular disparities exist in the mantis brain, we recorded intracellularly in the optic lobe, the major visual processing centre in insects (Fig. 1a, b). Animals viewed a computer screen through coloured filters enabling us to control stimuli to each eye separately and thus presenting images in 3D^7^ (Fig. 1a). Neurons were stained for subsequent identification.

We find that the praying mantis brain harbours at least four classes of neuron that are tuned to binocular disparities. These are the first neurons discovered in any invertebrate with properties suitable for supporting stereoscopic vision. The binocular response fields of several neurons show clear evidence of centre-surround mechanisms and are similar to disparity-tuned neurons in the vertebrate visual cortex.

## Results

### Mantis neurons tuned to binocular disparity

We identified a tangential projection neuron of the optic lobe, TAOpro, which is well suited to detect stereoscopically-defined mantis prey. We recorded from this neuron type only a single time. It ramifies in both outer lobes and the most distal layer of the anterior lobe of the lobula complex (LOX), a highly structured visual neuropil in the mantis brain^8^. The neuron projects to the ventromedial protocerebrum into what corresponds to the vest and/or the posterior slope in other insects^9,10^ (Fig. 1c). The TAOpro-neuron ramifications within the LOX covered large areas but were concentrated in the more ventral regions (Fig. 1c). In ventral parts of outer lobe 1, the neuron covered the whole inner surface from posterior to very frontal. We recorded TAOpro’s responses to a spiralling disc stimulus (Fig. 1d) which mimics mantis prey, i.e. a small, dark item that moves in front of a bright background^11^. During behavioural experiments mantids readily strike at the disc when its disparity indicates it is in catch range, but not in the control condition with reversed disparity^7^. When the same stimulus was presented to the restrained praying mantis during neuronal recording, the TAOpro-neuron responded vigorously for the disparity indicating catch range, and only weakly for the control condition (Fig. 1e; Wilcoxon rank sum test, *p* = 5.2 × 10^−4^).

To understand the neuronal computation supporting this response, we used our main stimulus comprising a vertical bar, 13° wide and flashed briefly at six different, non-overlapping locations independently in each eye (Fig. 1f and Methods). Vertical bars avoided the need to identify receptive field elevation while enabling us to vary horizontal disparity. For studying potential prey-detector neurons, we used dark bars on a brighter background, since mantids strike preferentially at dark prey^11^. Each eye saw either a single bar or a blank screen. In this way we simulated virtual objects at a range of 3D locations in front of the animal (Fig. 1f), as well as control locations not corresponding to any single location in space (Supplementary Fig. 1).

**Fig. 2:**
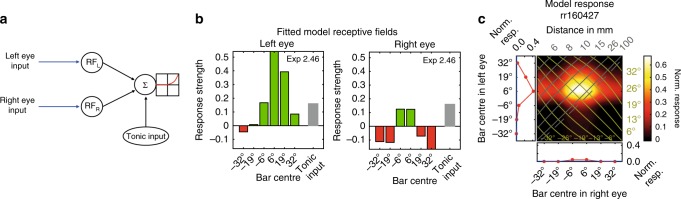
Linear-nonlinear model. **a** Outline of linear-nonlinear model components. Visual inputs are filtered by left-and right-eye receptive fields, then summed linearly along with tonic input, followed by spiking threshold and power-law. **b** Fitted left and right receptive fields for TAOpro-neuron; green = excitation, red = inhibition, grey = tonic input (shown in both RF plots, but applied only once), exponent in upper right corner (exp). **c** Fitted model responses for TAOpro-neuron

Tested monocularly, the TAOpro-neuron responded only to bars presented in the left eye (Fig. 1g). However, the single blob-like peak in the binocular response field shows that it receives binocular input. If there were input from only the left eye, the binocular response plot would show an excitatory horizontal stripe instead of a clear peak. Stimuli presented towards the periphery of the right eye must provide inhibition which serves to shape the excitatory input provided by the left eye. This response resembles disparity-tuned simple cells in the mammalian cortex^12^ and means that the neuron responds selectively to a combination of object locations in left and right eyes, and thus to a particular location in 3D-space. The response peaked for left-eye stimulation at 6° and right-eye at 0°, corresponding to an object located ~3° to the right of midline at a distance of ~50 mm (cf azimuth/distance isolines Fig. 1f)—an ideal strike target location for mantises^13^. For other locations, the response is much weaker, because input from the right eye provides an inhibitory surround which acts to suppress the neuron’s response to stimuli closer than 15 mm or further away than 100 mm (Fig. 2b, right panel). Similar inhibitory surrounds were found in other neuron types (see below).

In vertebrate stereopsis, disparity-tuned simple cells are well described by a linear-nonlinear (LN) model^12^ in which each eye’s image is filtered by a linear receptive field, and the result summed and passed through a threshold-plus-power-law non-linearity (Fig. 2a). We fitted this model to our mantis neuron responses. The fitted parameters were the outputs of the monocular receptive fields in response to a bar at each of the 6 locations in each eye, plus any tonic input and the exponent of the non-linearity (see Methods). The model accounted well for the response of TAOpro (Fig. 2c) and most other neurons we investigated (see below and Table 1).

**Table 1:** Responsiveness of neurons to left and right eye bar stimulation

Neuron class	Neuron ID	Input → output	Stimulus type	Repetitions	ANOVA	F-values (L/R/L* R)	*p*-values (L/R/L* R)	Variance explained	Species
TAOpro	rr160427	Left OL → CB	Dark bars (on)	18	L, R, I	100.79/16.67/3.73	0/0/0	96%	*H*
			Bright bars (on)	3	L, ns, ns	13.83/1.76/0.89	0/0.115/0.651	88%	
COcom	rr151123	Left OL → right OL	Dark bars (on)	11	L, R, I	93.04/5.33/2.93	0/0/0	90%	*H*
COcom	rr160127	Right OL → left OL	Dark bars (on)	10	L, R, I	7.76/6.24/2.14	0/0/0.0002	86%	*H*
COcom	rr160708	ND	Dark bars (on)	11	L, R, I	21.38/23.47/2.43	0/0/0	86%	*H*
COcom	rr160214	Right OL → left OL	Dark bars (on)	29	ns, ns, ns	1.88/0.36/1.35	0.0813/0.905/0.0814	21%	*H*
			Bright bars (on)	12	ns, R, ns	0.13/2.95/0.72	0.993/0.0076/0.888	30%	
COcom	rr170117	Right OL → left OL	Dark bars (on)	10	L, R, I	4.51/76.63/2.02	0.0002/0/0.0006	91%	*R*
COcom	rr171019	Right OL → left OL	Dark bars (on)	13	L, R, ns	2.67/26.73/1.05	0.0145/0/0.3948	80%	*H*
			Bright bars (on)	4 (0.2 nA inj)	ns, ns, ns	1.45/1.43/1.35	0.198/0.208/0.108	33%	
TAcen	rr161114	CB → left OL	Bright bars (on)	15	L, R, ns	39.08/69.01/0.65	0/0/0.946	97%	*R*
			Dark bars (on)	12	L, R, ns	4.67/3.12/1.43	0.0001/0.0052/0.0524	36%	
			Dark bars (off)	12	L, R, ns	162.31/40.94/1.26	0/0/0.150	97%	
TAcen	rr170213	CB → left OL	Bright bars (on)	12	L, R, I	3.83/94/1.68	0.001/0/0.009	88%	*R*
			Dark bars (on)	4 (0.4 nA inj)	ns, R, ns	0.89/2.68/1.1	0.504/0.0171/0.337	38%	
			Dark bars (off)	4 (0.4 nA inj)	ns, R, ns	1.39/23.53/0.76	0.223/0/0.836	81%	
TAcen	rr170606	CB → left OL	Bright bars (on)	10 (some with 0.1 nA inj)	L, R, I	249.17/21.76/2.16	0/0/0.0002	97%	*H*
			Dark bars (on)	2 (0.1 nA inj)	L, R, ns	8.56/7.39/0.91	0/0/0.6135	74%	
			Dark bars (off)	2 (0.1 nA inj)	L, R, ns	42.85/5.8/0.59	0/0.0001/0.949	93%	
TAcen	rr170628	CB → left OL	Bright bars (on)	17	L, R, ns	26.21/23.21/0.66	0/0/0.936	92%	*R*
			Dark bars (on)	13 (some with 0.3 nA inj)	ns, R, ns	0.87/5.95/0.56	0.5176/0/0.9832	61%	
			Dark bars (off)	13 (some with 0.3 nA inj)	L, R, ns	16.68/3.27/0.73	0/0.0036/0.8815	79%	
TAcen	rr170403	CB → left OL	Bright bars (on)	11	L, R, ns	61.34/16.6/0.92	0/0/0.604	94%	*H*
TAcen	rr160106	CB → left OL	Dark bars (on)	13	L, R, ns	9.3/4.59/0.69	0/0.0001/0.912	74%	*H*
			Dark bars (off)	13	L, R, ns	23.35/39.57/0.79	0/0/0.802	93%	
TMEcen	rr160201	CB → left OL	Bright bars (on)	32	L, R, I	6.45/6.1/1.49	0/0/0.033	58%	*H*
			Dark bars (on)	15	ns, R, ns	0.7/2.22/0.83	0.647/0.0391/0.752	26%	
TMEcen	rr160818	CB → left OL	Bright bars (on)	13	L, R, I	23.18/30.26/2.1	0/0/0.0003	82%	*H*
			Dark bars (on)	15	L, R, I	86.25/60.58/1.68	0/0/0.0082	94%	
TMEcen	rr161025	CB → left OL	Bright bars (on)	15	L, R, I	6.49/189.94/1.54	0/0/0.0247	95%	*H*
TMEcen	rr170723	CB → left OL	Dark bars (on)	23 (0.15 nA inj)	L, R, I	334.38/25.71/2.96	0/0/0	97%	*R*

Sixth column indicates whether there was a significant (*p* < 0.05) main effect of left (L) or/and right (R) eye stimulation on neuronal response as tested with two-way-ANOVA (see Methods: Statistical analysis). A significant interaction term in the two-way-ANOVA is indicated by “I”, non-significance by ‘ns’. Degrees of freedom were 6 for left and right eye main effects, respectively and 36 for interaction terms. F —and *p*-values are provided in 7th and 8th column, respectively. *p*-values smaller than 0.0001 are given as 0. Recordings were usually done without concurrent depolarizing current injection unless indicated by ‘inj’ with the amount of current indicated in nA. On-responses were evaluated in a 250 ms time window starting 1 ms after stimulus onset. Off responses were evaluated in a 200 ms time window starting 50 ms after stimulus off. Abbreviations: *H*, *Hierodula membranacea*; L left eye, I interaction (L*R), ND not determinable (no clear peak response), ns not significant (*p* > 0.05), OL optic lobe, CB central brain, R right eye, *R*, *Rhombodera megaera*

**Fig. 3:**
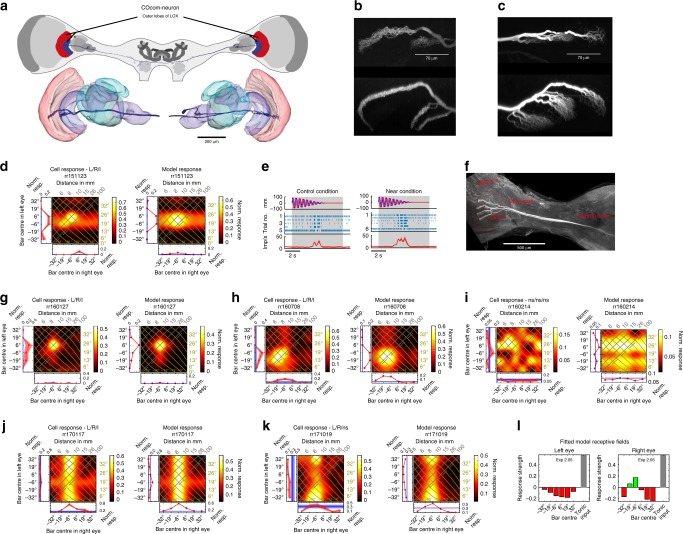
Columnar, commissural neuron, connecting both lobula complexes. **a** COcom-neuron (columnar commissural neuron of the outer lobes) reconstruction, anterior view. Neuron ramifies in outer lobes of both LOX and in central brain. **b**, **c** Maximum projections of confocal horizontal (posterior to anterior orientation; top) and frontal sections (dorsal to ventral orientation; bottom) through outer lobe 1 and 2 in right (**b**) and left (**c**) optic lobe. Terminal neurites are strongly beaded in right (**b**) and smoother in left optic lobe (**c**). **d** Measured (left) and modelled (right) response fields for neuron in (**a**, **b**, **c**) for flashed dark bars. **e** response of same neuron to spiralling disc. **f** Maximum projection of confocal microscopy slices through left side of praying mantis brain (posterior view), showing several COcom-neurons stained during the experiment that culminated in the recording of neuron rr170117 (response in j). OLO1 outer lobe 1 of LOX, OLO2 outer lobe 2 of LOX. **g**–**k** as **d** for five further COcom-neurons (see Supplementary Fig. 3 for fitted receptive fields and responses to bright bars for two neurons). Response field headers provide outcome of two-way-ANOVA with “L” (“R”) being significant left (right) eye input and “I” significant interaction term (see Table 1), otherwise “ns” meaning not significant. **l**, Fitted receptive fields for neuron in (**k**)

A second neuron type which we identified, a columnar commissural neuron, *COcom*, seems homologous to *Drosophila* LC14-neurons^14^. Similar to LC14-cells the type COcom comprises an array of brain-spanning neurons extending from one optic lobe to the other (Fig. 3a, f). COcom-neurons have recently been discovered to be tuned to small moving objects^15^. They possess beaded (i.e. output^16^) ramifications contralateral to the cell’s soma, but smooth (input^16^) endings ipsilaterally (Fig. 3b, c; Table 1); on both sides these ramifications are narrow dorsoventrally and very wide anteroposteriorly, potentially spanning the entire outer lobe. We recorded from six of these cells in six different animals. Where COcom-neurons have a clear dominant eye, visible as a horizontal or vertical stripe in the binocular response field (Fig. 3d, j, k), this is always the putative input side. COcom-neurons also send output fibres to the central brain (Fig. 3a).

Five of the six COcom-neurons were clearly binocular (significant main effect of both left and right-eye stimulation, Fig. 3d, g, h, j, k; Table 1) and four of these also showed significant binocular interaction (Fig. 3d, g, h, j). Three neurons (Fig. 3d, g, h) had well-localised excitatory peaks for a preferred 3D location. In each case these peaks were at 15–100 mm distance and within 20° eccentricity, a region of space where behavioural experiments^6^ have shown that mantis stereopsis operates for prey capture. These were again well modelled by combining binocular excitation at the preferred location with inhibition in peripheral regions (Supplementary Fig. 3a, b, c). In two neurons, bars in this region elicited less excitation than the optimal monocular bar in the right eye (Fig. 3j, k), possibly because of inhibition by input from the left eye. In vertebrates such cells are known as tuned-inhibitory neurons, in contrast to tuned-excitatory neurons whose receptive fields have a similar structure in both eyes^17^. The neuron from Fig. 3a–d also showed significant disparity tuning to the spiralling disc stimulus (Wilcoxon rank-sum test *p* = 0.0043, Fig. 3e; for disc responses of the remaining COcom-neurons see Supplementary Fig. 4).

### Disparity sensitive feedback neurons

**Fig. 4:**
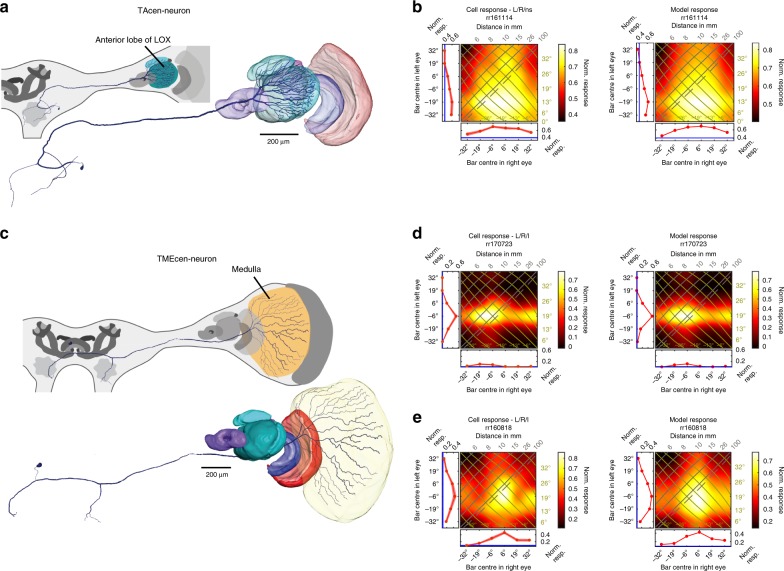
Centrifugal (feedback) neurons. **a** Anterior view of reconstructed TAcen-neuron (tangential centrifugal neuron of the anterior lobe) with ramifications in anterior lobe of LOX and central brain. **b** Cell (left) and model (right) response fields for an example TAcen-neuron to flashed, bright bars (responses of 5 further TAcen-neurons in Supplementary Figs. 6, 7). **c** Anterior view of reconstructed TMEcen-neuron (tangential centrifugal neuron of the medulla) with ramifications in medulla and central brain. Scale bar 200 µm. **d**, **e** Cell and model responses for two TMEcen-neurons to flashed dark bars. Four further TMEcen-neuron response fields in Supplementary Figs. 8c–f. Response field header with outcome of two-way-ANOVA with “L” (“R”) being significant left (right) eye input and “I” significant interaction term (see Table 1), otherwise “ns” meaning not significant

The morphology of two additional, disparity-sensitive neuron types suggests that they convey information centrifugally (soma in central brain—Fig. 4a,c; beaded terminal neurites in optic lobe—Supplementary Fig. 5) from the central brain to the LOX (TAcen-neurons) and the medulla (TMEcen-neurons). TAcen-neurons generally responded more strongly to bright than to dark bars (Fig. 4b, Supplementary Figs. 6, 7). TAcen-neurons have broad excitatory receptive fields with peak responses for far distances or even diverging lines of sight.

The four TMEcen-neurons which we recorded were also binocular (Fig. 4d, e; Supplementary Fig. 8). However, unlike TAcen-neurons, different TMEcen-neurons responded to different disparities and even contrasts, comprising preferences for dark objects in catching range (Fig. 4d) or far away (Fig. 4e) and bright objects at diverse distances (Supplementary Fig. 8c, d, f).

## Conclusions

**Fig. 5:**
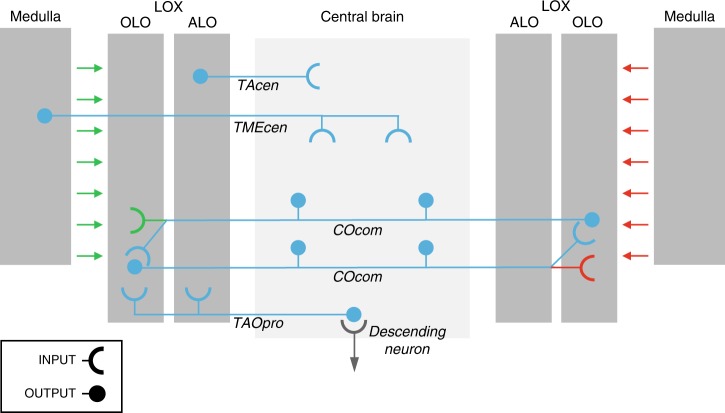
Summary of neurons sensitive to binocular disparity in the praying mantis brain. Grey shaded areas represent brain regions with ramifications of the recorded neurons; optic lobe regions are shaded darker. Feedforward visual information flow from left eye is illustrated in green, from right eye in red and combined, binocular blue. Input regions are shown with semi-circles, output with disks. For clarity TAOpro, TMEcen and TAcen are shown only on left side although we envisage symmetrical neurons also on the right, and only those parts of the lobula complex that harbour neuron ramifications are shown. LOX lobula complex, ALO anterior lobe of the LOX, OLO outer lobe of the LOX, COcom commissural columnar neuron of the outer lobes, TAcen centrifugal tangential neuron of the anterior lobe, TAOpro tangential projection neuron of the anterior and outer lobes, TMEcen centrifugal tangential neuron of the medulla

Figure 5 summarises the four neuronal classes presented above. These are the first neuronal correlates of insect stereopsis. Further work will be required to establish a complete circuit, but a working hypothesis consistent with our data is that disparity sensitivity is established in the LOX by COcom-neurons which receive visual input from one eye in the ipsilateral optic lobe and from the other eye via contralateral COcom-neurons (Fig. 5). A similar circuit was recently suggested for brain-spanning neurons in the crab^18^. TAOpro is anatomically well positioned to deliver the signal for the raptorial strike, since it projects to premotor regions where, in other insects^19–21^, descending neurons receive input that is relayed to thoracic motor centres. Physiologically, the excitatory centre/inhibitory surround structure observed in COcom and TAOpro receptive fields is consistent with the size tuning for prey objects shown in behavioural experiments^11,13^. TAcen and TMEcen-neurons deliver disparity feedback to the optic lobes, presumably to modulate visual information processing (Fig. 5). TAcen-neurons have recently been found to be sensitive to wide-field motion^15^, and we now find that they respond best to bright stimuli at very far distances: all properties associated with the background against which prey appears. Thus, TAcen-neurons could aid the segregation of objects from background. This was proposed for CH-cells in the blowfly^22^, which also project from central brain to LOX. Finally, TMEcen-neurons relay disparity information from the central brain to the medulla, the early (second) visual neuropil. Here TMEcen-neurons could either boost overall neuronal processing when relevant stimuli occur, or they might even guide attention in 3D-space. Insect centrifugal neurons have been repeatedly shown to modulate visual processing including involvement in selective attention^23–27^.

Our work establishes that numerous disparity sensitive neurons are indeed present in the praying mantis brain, ruling out previous suggestions that insect stereopsis may be extremely simple with disparity being computed only at a single, later motor output stage^5,6^. Rather, we have identified neurons which are tuned to different locations in 3D-space, at a range of distances and horizontal eccentricities (Supplementary Fig. 9), confirming disputed deductions from behavioural data^6,28^. A linear/nonlinear-model like those proposed to explain disparity selectivity in simple cells of the vertebrate visual cortex^12^ also captures the behaviour of most mantis neurons. Binocular disparities are computed early in the visual pathway, in the LOX, and are fed back even earlier, to the medulla. Such disparity feedback has not yet been identified in any other species. It may be an adaptation associated with the particular demands of insect stereopsis—and thus valuable for machine stereo in similar applications—or it may turn out to be widespread, including in our own brains. Thus, insect stereopsis suggests new approaches to machine stereo vision and demands new reflection regarding stereo vision in humans.

## Methods

### Animals

Experiments were carried out on 17 large adult praying mantids of the species *Hierodula membranacea* and *Rhombodera megaera* with an interocular distance of about 8 mm. Animals were housed in individual containers at a temperature of 25 °C and a 12 h light/dark cycle. Adult animals were fed with a live cricket twice and younger mantids three times a week.

### Animal preparation

Animals were mounted on custom-made holders with BluTack® and wax; their mouthparts were removed, and their head was immobilized by wax. A hole was cut into the posterior head capsule to allow access to the brain. Fat and muscle tissue surrounding the brain were removed. The neural sheath was stripped away at the region where the recording electrode was inserted. The gut was removed within the head capsule and prevented from leaking within the thorax by ligating it. A wire platform supported the brain from anterior to further stabilize it. During recording of neural activity the brain was submerged in cockroach saline.

### Neuronal recordings

All recordings were performed exclusively in the left optic lobe. We expect the same set of neurons is present on both sides of the brain. We recorded intracellularly with sharp electrodes from 17 neurons. Each cell was recorded in a different animal. All neurons are listed in Table 1. Thirteen neurons had ramifications in the LOX and four had ramifications in the medulla. The neurons were identified by stainings with neuronal tracer (see below). Microelectrodes were drawn from borosilicate capillaries (1.5 mm outer diameter, Hilgenberg, Malsfeld, Germany) on a microelectrode puller (P-97, Sutter Instrument, Novato, CA). Electrode tips were filled with 4% Neurobiotin (Vector Laboratories, UK) in 1 M KCl and their shanks with 1 M KCl. The electrodes had tip resistances of 70–150 MOhm. Signals were amplified (BA-03X amplifier; NPI), digitized (CED1401 micro; Cambridge Electronic Design, UK), and stored using a PC with Spike2 software (Cambridge Electronic Design, UK). About 0.1–1 nA of depolarizing current was applied for several minutes to iontophoretically inject Neurobiotin immediately after recording and in some recordings in-between the stimulus sequences. We only injected and analysed those neurons for which we could acquire responses to the presentation of at least 10 repetitions of the bar stimulus.

### Histology

After neuronal recordings animal heads were fixed overnight in a mixture of 4% paraformaldehyde, 0.25% glutaraldehyde, and 0.2% saturated picric acid in 0.1 M phosphate buffer. Afterwards brains were dissected out of the head capsule. The labelled neurons were made visible for confocal laser scanning microscopy (Leica TCS-SP5/SP8; Leica Microsystems) by treatment of the brains with Cy3-conjugated streptavidin (Dianova, Hamburg, Germany). More specifically after incubation with the fixative, brains were first washed with 0.1 M PBS and then with 0.1 M PBS containing 0.3% Triton X-100. Afterwards the brains were incubated with streptavidin-Cy3 for 3 days at 4 °C. Then the brains were again washed in PBS before dehydrating them in an ethanol series (25, 50, 70, 90, 95, and 100%, 15 min each). Finally, the brains were cleared by first treating them with a solution of 50% ethanol and 50% methyl salicylate (20 min) and then with pure methyl salicylate (Merck, Darmstadt, Germany) until transparent (at least 60 min). As a last step the brains were mounted in Permount (Fisher Scientific, Pittsburgh, PA) between two glass cover slips which were separated by spacing rings to avoid compression.

### Visual stimulation

We used anaglyph technology^4,7^ to present 3D stimuli on a computer monitor (DELL U2413 LED). Tethered mantids watched the computer screen through spectral filters while we performed neuronal recordings in their brain. We presented stimuli with different colours (green and blue) that matched the spectral properties of the filters so that each eye saw only the image it was intended to see. We performed electroretinograms as described in ref. ^7^ to ensure same perceived brightness through both spectral filters by adjusting the brightness gain for both colour channels accordingly. The computer screen was positioned at a viewing distance of 10 cm from the praying mantis.

All stimuli were custom written in Matlab (Mathworks) using the Psychophysics Toolbox^29–31^. We presented two main stimuli for the current study. Most importantly we analysed monocular and binocular response fields of neurons with a flashed bar stimulus. For this we divided the region of binocular overlap into six non-overlapping vertical stripes of 12.8° horizontal and 99.5° vertical extent (Fig. 1f). In this way we covered almost 77° of the fronto-azimuthal visual field. This is slightly wider than the approximately 70° binocular overlap of praying mantids^6^. Bars were presented either to one eye only, for recording monocular response fields, or two bars concurrently, one for the left and one for the right eye, for determining binocular response fields.

We used bars instead of structures with smaller vertical extent because of the comparatively short recording times possible with sharp electrodes. In this way we avoided the need to identify receptive field elevation while enabling us to vary horizontal disparity, the difference in the bar’s location between left and right eyes. Because insect eyes are offset horizontally and fixed on the head, horizontal disparity along with visual direction specifies a unique 3D position in space^32,33^, as shown in Fig. 1f. All bar combinations, including both monocular and binocular conditions, were shown in pseudorandom order. The bars were displayed for 250 or 500 ms with a pause of the same duration in between each presentation. After all bar positions had been displayed a pause of 1.7–4.5 s followed, before the procedure started again. These stimulation times and pauses were chosen after preliminary experiments had shown that they sufficed our requirements for (1) being long enough to elicit strong responses and thus reliable response estimates, (2) not influencing successive stimulations and (3) still provide sufficient time to acquire at least 10 repetitions with at least one bar condition (providing dark or bright bars).

The second stimulus was similar to what was found earlier to be a very effective elicitor of the praying mantis prey capture strike^7^. A 22°-diameter dark disc in front of a bright background appeared peripherally and spiralled in towards the centre of the screen (Fig. 1d). On reaching the screen centre, after 5 s, it stayed there for 2 s before vanishing. Small quivering movements were superimposed on the principal spiral trajectory and in the final 2 s stationary disc phase. The disc was simulated to float at a distance of 25 mm in front of the praying mantis in order to simulate an attractive target in catch range of the animal. This was achieved by presenting one disc on the left hand side, which was only visible to the right eye and a disc of identical dimensions slightly shifted to the right, which was only visible to the left eye. We refer to this stimulus condition as the near condition. As a control condition, the left and right eye discs were swapped so that the right eye now saw the right hand side disc and the left eye saw the left hand side disc (cf Supplementary Fig. 1c vs d for equivalent bar stimulus).

### Microscopy and image data analysis

Whole mounts were scanned with confocal laser scanning microscopes (CLSM, TCS SP5 and SP8, Leica Microsystems, Wetzlar, Germany) with a 10× oil immersion objective lens (SP5) or a 10× or 20× dry lens (SP8). The detail scans of the neuritic endings in Fig. 3b and c were done with a 63er glycerol immersion objective lens and the SP5 microscope. The SP5 microscope was located in the Biology Department of Marburg University (Germany) and the SP8 microscope in the Bioimaging Unit at Newcastle University (UK).

Neuronal reconstructions were done with the SkeletonTree tool within Amira^34^. The reconstructed neurons were registered manually into a reference LOX^8^ in Amira 5.33. The schemes of the mantis brain were done in Adobe Illustrator CS5 (Adobe Systems, Ireland).

### Data evaluation

Data analysis was done in Matlab (The MathWorks, Natick, MA). Our analysis is based exclusively on spike counts because we usually did not observe postsynaptic potentials. The likely explanation is that our recording site was distant from dendritic input regions. We deduced that a stimulus must be having an inhibitory effect from the reduction in spike rates, as for example for the TAOpro-neuron in Fig. 1g during binocular stimulation.

Bar stimulus induced spike counts were determined in 250 ms time windows starting at time 1 ms when a bar was displayed. The background spike count was determined in 800 ms time windows preceding each stimulus sequence. Responses were converted to spiking rates per second and normalized by dividing by the highest spiking rate that occurred during either bar presentation or background firing, depending which one was higher. Afterwards the responses were averaged across identical stimulus conditions for each cell.

For several TAcen-neurons we also determined dark bar off-responses in a 200 ms time window starting 50 ms after the respective bar was switched off (Supplementary Fig. 7 and Table 1).

We interpolated all binocular response fields from 6 × 6 to 100 × 100 with the Matlab function imresize in bicubic mode. An example raw plot and its upsampled version is shown in Supplementary Fig. 2.

Neuron rr170403 was only weakly stained and it was not possible to trace the main neurite into the central brain. Moreover, in the confocal scan it was partly superimposed by a second even weaker stained projection neuron. We included rr170403 in our analysis, because we consider it most likely to belong to the TAcen-class of neurons as identified by its typical ramifications in the anterior lobe of the LOX.

### Statistical analysis

Responsiveness of neurons to left or right eye stimulation was determined by two-way-ANOVA (anova2-function in Matlab; requirement for significance *p* < 0.05). The two factors were the location of the bar in the left and right eye respectively. Each factor had seven levels, corresponding to the six possible bar locations plus the blank-screen condition. A significant main effect of each factor therefore means that the response differed between at least two different bar positions for the respective eye, and/or the response differed for at least one bar location from the spontaneous rate. A non-significant interaction term means that binocular response was well described by the sum of monocular responses; a significant interaction means that they combine non-linearly.

Responsiveness to the spiralling disc stimulus was determined for a selection of neurons via two-sided Wilcoxon rank sum test (Matlab ranksum-function; *p* < 0.05) by comparing spike counts within a time window of 3.5 s, starting 1.5 s after stimulus onset, between the near and control conditions.

### Modelling

For simulating response fields we applied a LN model used for modelling simple cell responses in vertebrate stereopsis (the simple cell model in ref. ^12^, generalised to allow arbitrary receptive fields and output exponent). The model assumes that visual stimulation contributes excitatory or inhibitory input dependent on the eye and location of the stimulation; that is, the model contains receptive fields for both the left and the right eye (Fig. 2a). The inputs from both eyes are filtered by the receptive field and then summed linearly along with a tonic input, necessary to account for a non-zero background rate in some neurons. If the result is negative or zero, the mean response is zero. If the result is positive, the mean response is given by its value raised to some exponent. The value of the exponent, the tonic input, and the monocular responses of the left and right eye receptive fields to bars in each of the six positions, together form 14 model parameters which we fitted to the mean neuronal response in 49 conditions (no visual stimulation, 12 monocular conditions and 36 binocular). We use the term response field to mean the measured average spiking rate of the neuron to bar stimuli at the specified location; we keep the term receptive field to refer to the linear part of the function governing this response, which was determined by the model fitting procedure.

### Reporting summary

Further information on research design is available in the Nature Research Reporting Summary linked to this article.

## Supplementary information

Supplementary Information

Reporting Summary

## Data Availability

Reconstructed neurons are available through NeuroMorpho.Org under 10.13021/ay7p-fw49 and neurophysiological data under 10.25405/data.ncl.8063327.

## Code availability

Code for evaluating the provided data and for generating figures is available under 10.25405/data.ncl.8063327.
