# Supplementary material for: A neuronal correlate of insect stereopsis
Source: Nat Commun. 2019 Jun 28;10:2845. doi: 10.1038/s41467-019-10721-z (PMC6599392; doi:10.1038/s41467-019-10721-z)
Supplement: Supplementary file 1 — Supplementary Information [file 41467_2019_10721_MOESM1_ESM.pdf]

Supplementary Information

A neuronal correlate of insect stereopsis

Rosner et al.

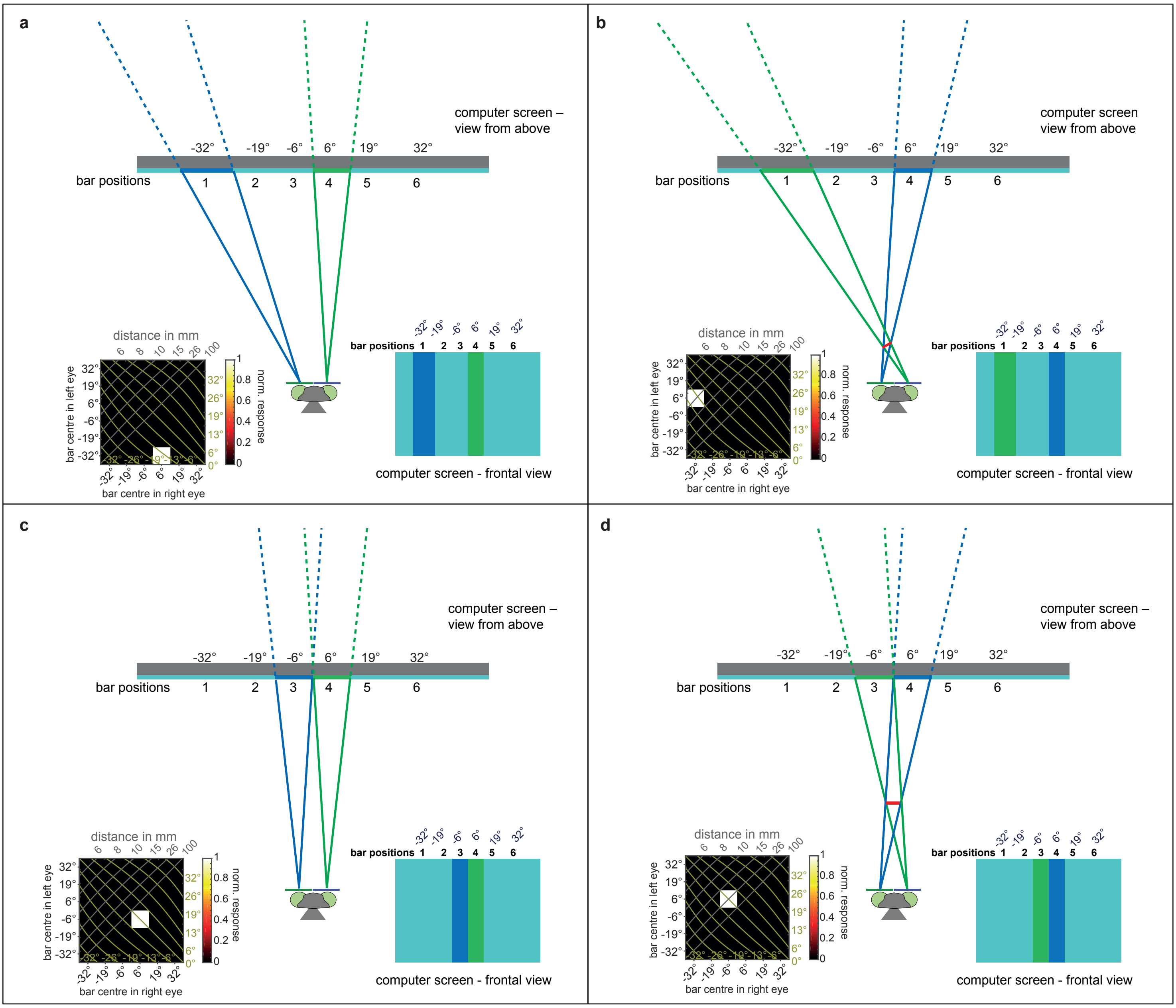

Supplementary Figure 1 | Visual stimulation and response field plotting in detail. a-d, Praying mantis with anaglyph filters watches computer screen with bar stimulus. Bottom left - binocular response field plot with neuronal response as would be observed if neuron responded exclusively to the shown stimulus configuration. Figures are not to scale. **a,c,** Lines of sight for left and right eye bar don't intersect in front of screen ("uncrossed disparity", control condition). These stimulus configurations are represented by points in the lower-right half of the binocular response plots. **b,d,** Lines of sight for left and right eye bar do intersect in front of screen ("crossed disparity", near condition). These stimulus configurations correspond to a bar located in front of the screen (shown in red) and are represented by points in the upper-left half of the binocular response plots.

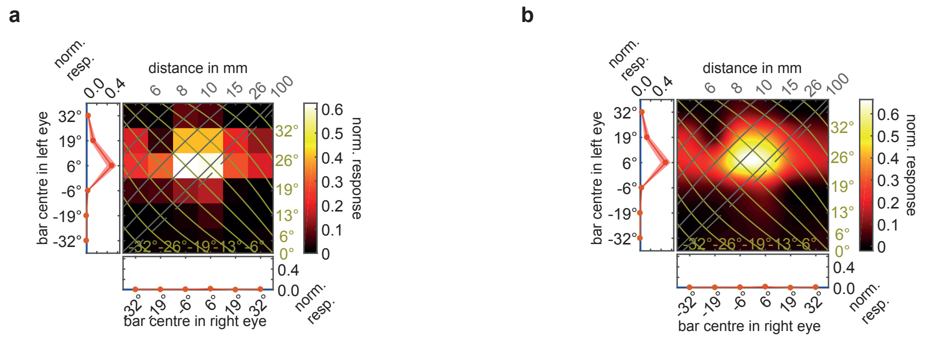

Supplementary Figure 2 | Raw and interpolated response field plots for TAOpro-neuron. a, Raw (non-interpolated) monocular and binocular response field plots. **b,** Interpolated binocular response field plot and non-interpolated, monocular response field plots.

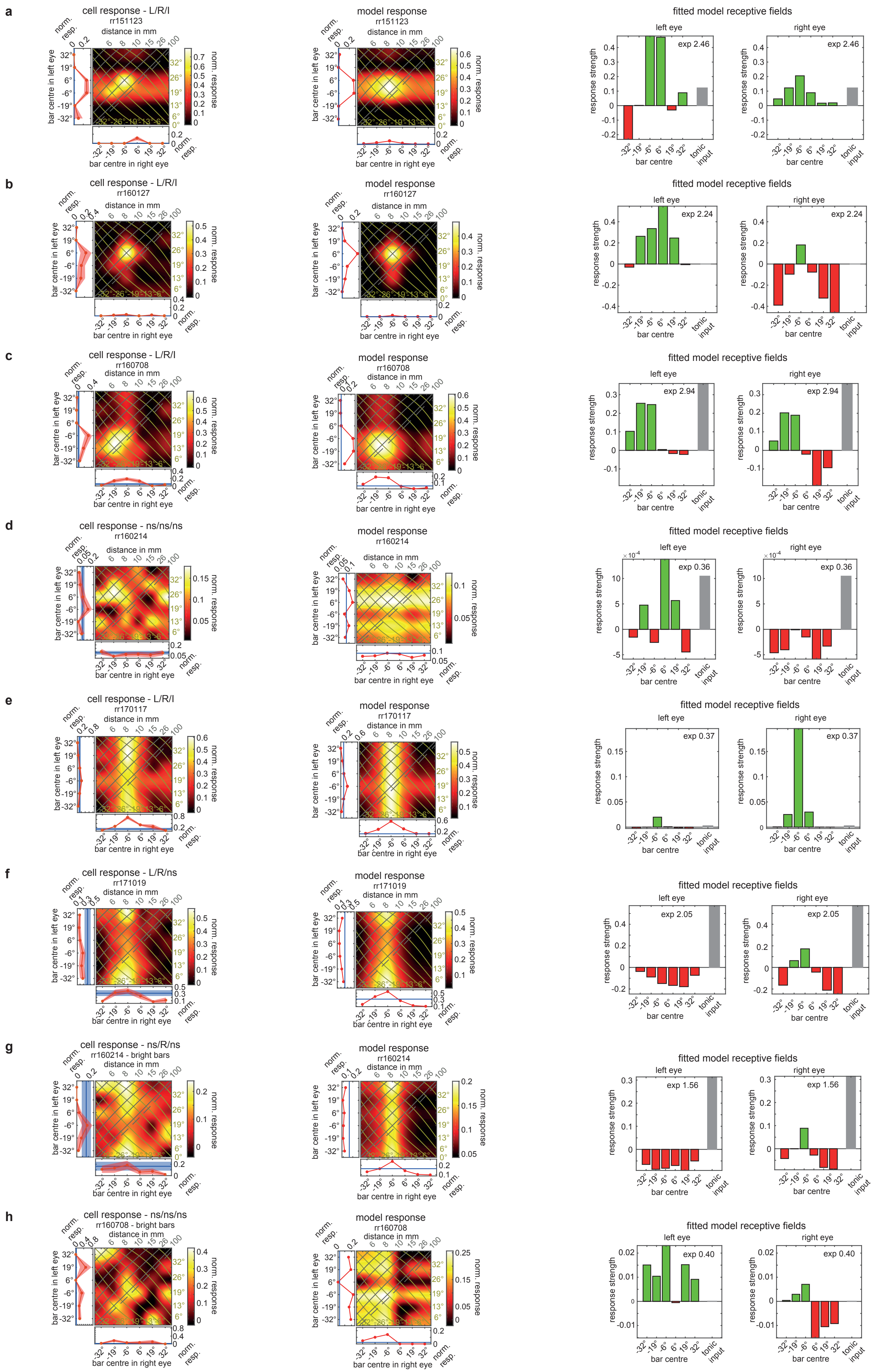
a
cell response - L/R/l
rr151123
model response
rr151123
fitted model receptive fields
left eye
right eye
exp 2.46
exp 2.46
b
cell response - L/R/l
rr160127
model response
rr160127
fitted model receptive fields
left eye
right eye
exp 2.24
exp 2.24
c
cell response - L/R/l
rr160708
model response
rr160708
fitted model receptive fields
left eye
right eye
exp 2.94
exp 2.94
d
cell response - ns/ns/ns
rr160214
model response
rr160214
fitted model receptive fields
left eye
right eye
exp 0.36
exp 0.36
e
cell response - L/R/l
rr170117
model response
rr170117
fitted model receptive fields
left eye
right eye
exp 0.37
exp 0.37
f
cell response - L/R/ns
rr171019
model response
rr171019
fitted model receptive fields
left eye
right eye
exp 2.05
exp 2.05
g
cell response - ns/R/ns
rr160214 - bright bars
model response
rr160214
fitted model receptive fields
left eye
right eye
exp 1.56
exp 1.56
h
cell response - ns/ns/ns
rr160708 - bright bars
model response
rr160708
fitted model receptive fields
left eye
right eye
exp 0.40
exp 0.40
distance in mm
bar centre in left eye
bar centre in right eye
norm. resp.
norm. response
response strength
bar centre
tonic input

Supplementary Figure 3 | Response field plots and fitted receptive fields for COcom-neurons. a-f, Left panels show monocular and binocular response field plots for all recorded COcom-neurons as presented in Fig. 3. The recordings were made during presentation of a dark bar to the left and/or the right eye. Response field headers state neuron ID and outcome of two-way-ANOVA with “L” (“R”) being significant left (right) eye input and “I” significant interaction term (see Table 1), otherwise “ns” meaning not significant. Middle panels show response field plots for model predictions. Right panels show fitted receptive fields for left and right eye with excitations (green bars) and inhibitions (red bars) at corresponding azimuthal locations (x-axis). Grey bars show tonic input (shown in both RF plots but applied only once). Exponent in upper right corner (exp). Negative (positive) values on x-axis indicate locations left (right) of centre. **g,h,** As a-f except that recordings were done during stimulation with bright instead of dark bars (for further information see also Table 1).

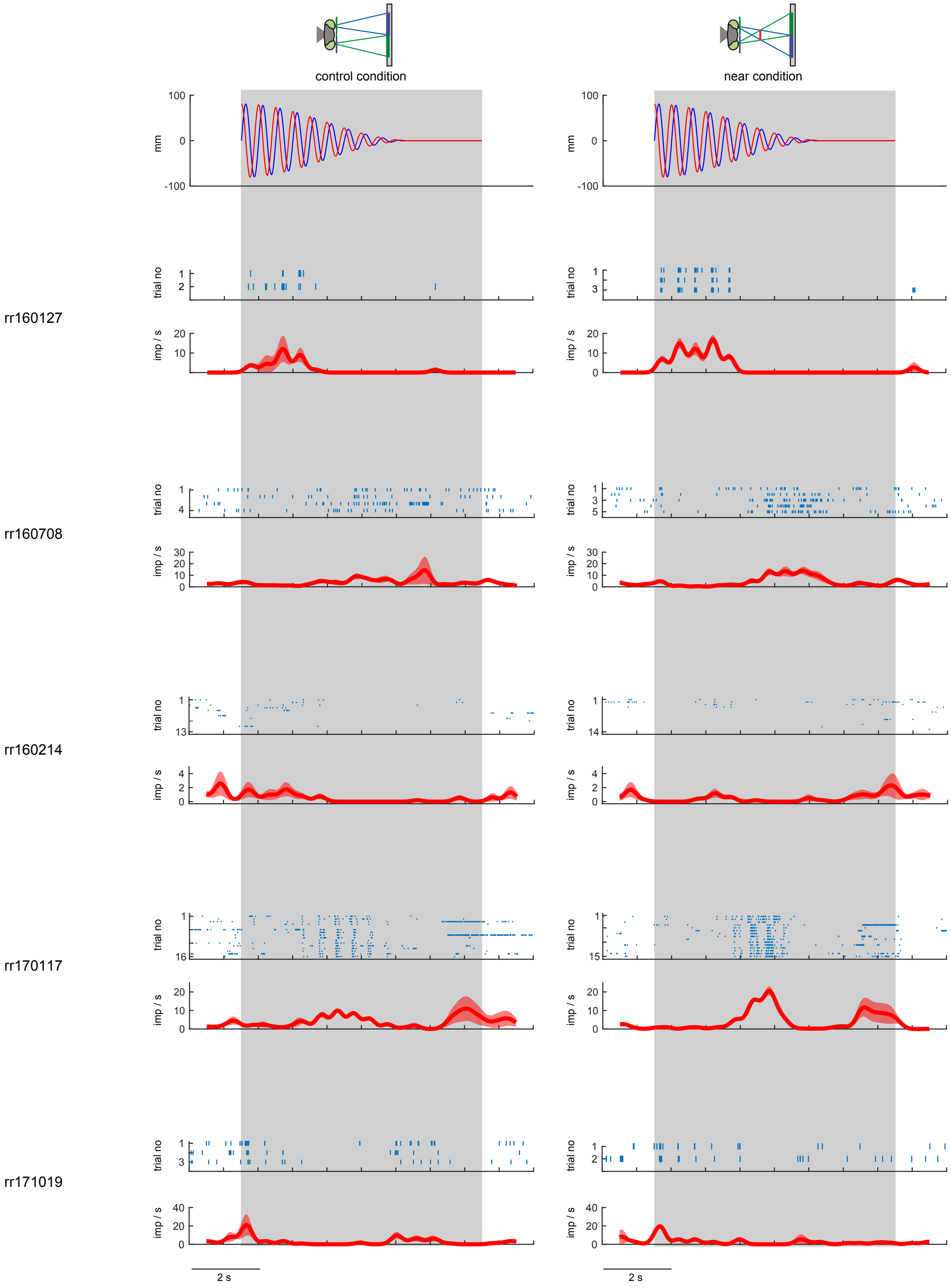

Supplementary Figure 4 | Responses to spiralling disc stimulus (grey shaded time period) of five COcom-neurons. Cell identities are given on left hand side. Sketches above show the stimulus disparity. Upper lane: vertical (blue) and horizontal (red) distance of disc from screen centre as function of time; negative values are left and lower side of screen. Lanes below show raster plots and spiking rates (average red line, ±1SEM ribbons) after Gaussian smoothing with SD of 150 ms for each of the cells. Right plots: virtual disc at 25 mm (in catch range), left: control (right and left eye disc swapped).

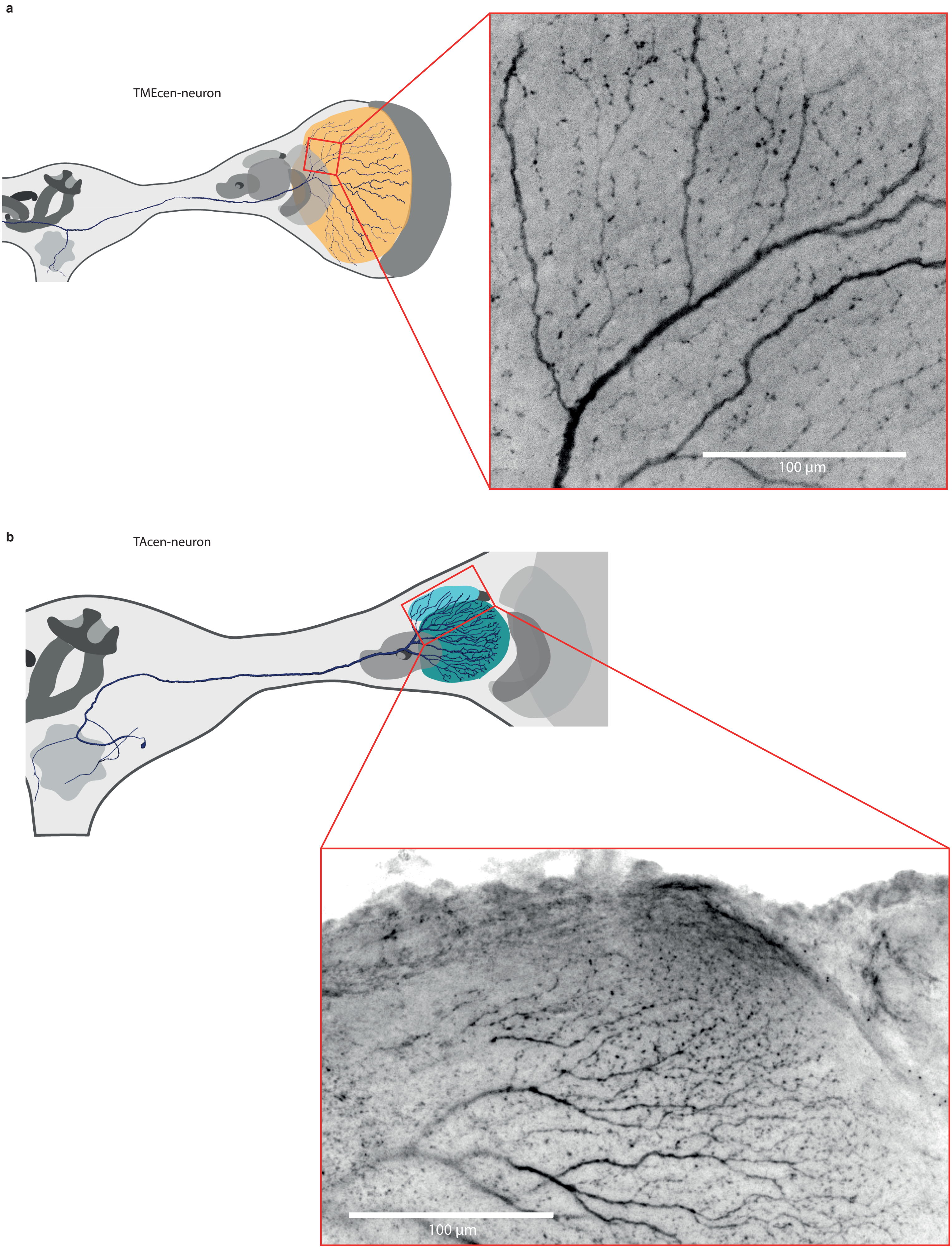

Supplementary Figure 5 | TAcen- and TMEcen-neurons have output arborizations in the optic lobe. a,b, Projection views of multiple confocal images for TMEcen-neuron (**a,** right) and TAcen-neuron (**b,** bottom right) branchings in left optic lobe. Location of recording windows indicated in left hand side schemes. In both neurons the terminal neurites are beaded/globular, indicating presynaptic regions[1].

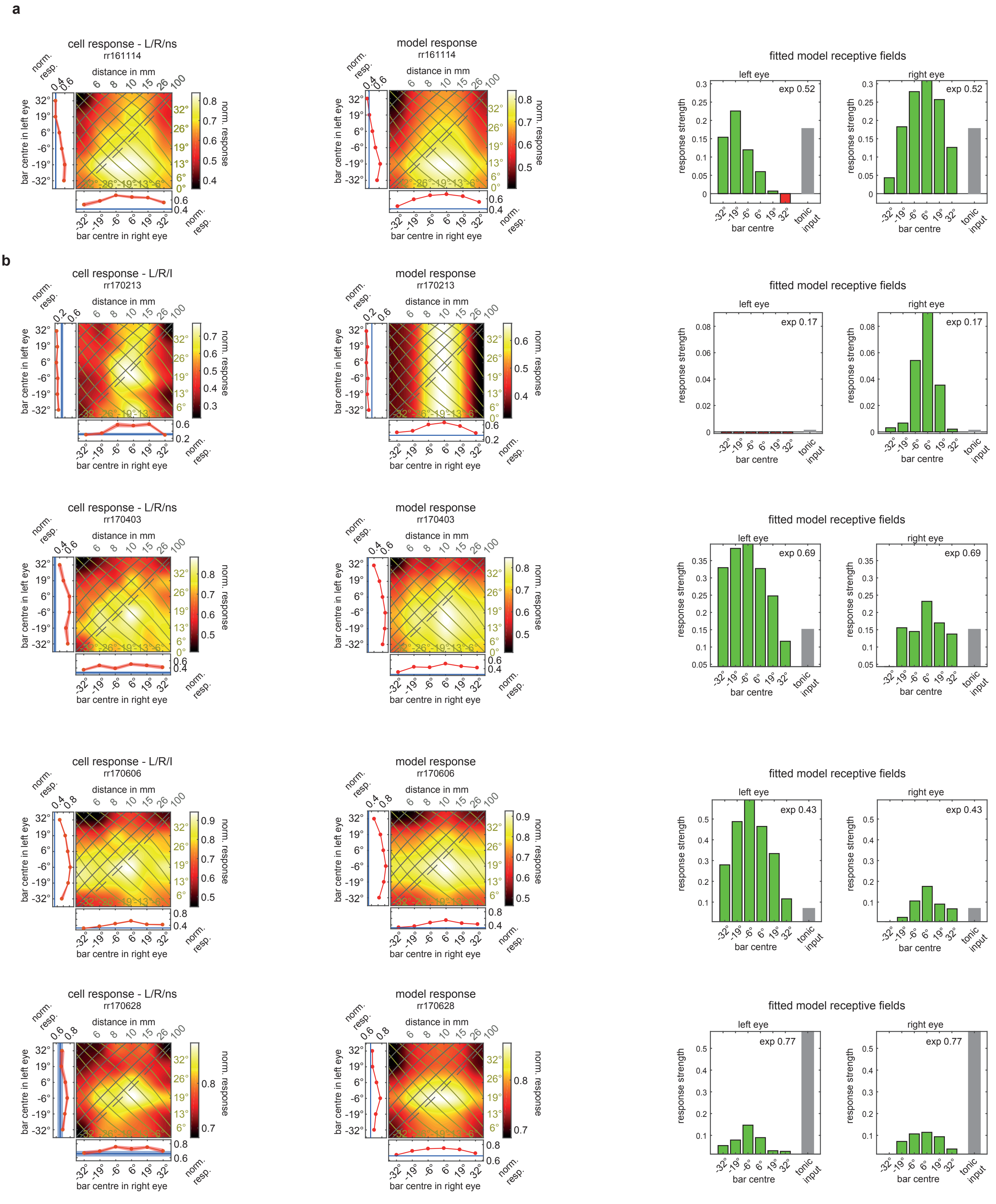

Supplementary Figure 6 | TAcen-neuron responses to flashed bright bars and model responses. a-e, Left panels show response field plots for TAcen-neuron bright bar stimulation. Response field headers state neuron ID and outcome of two-way-ANOVA with "L" ("R") being significant left (right) eye input and "I" significant interaction term (see Table 1), otherwise "ns" meaning not significant. Middle panels show raw response field plots for model predictions. Right panels show fitted receptive fields for left and right eye with excitations (green bars) and inhibitions (red bars) at corresponding azimuthal locations (x-axis). Grey bars show tonic input (shown in both RF plots but applied only once). Exponent in upper right corner (exp). Negative (positive) values on x-axis indicate locations left (right) of centre.

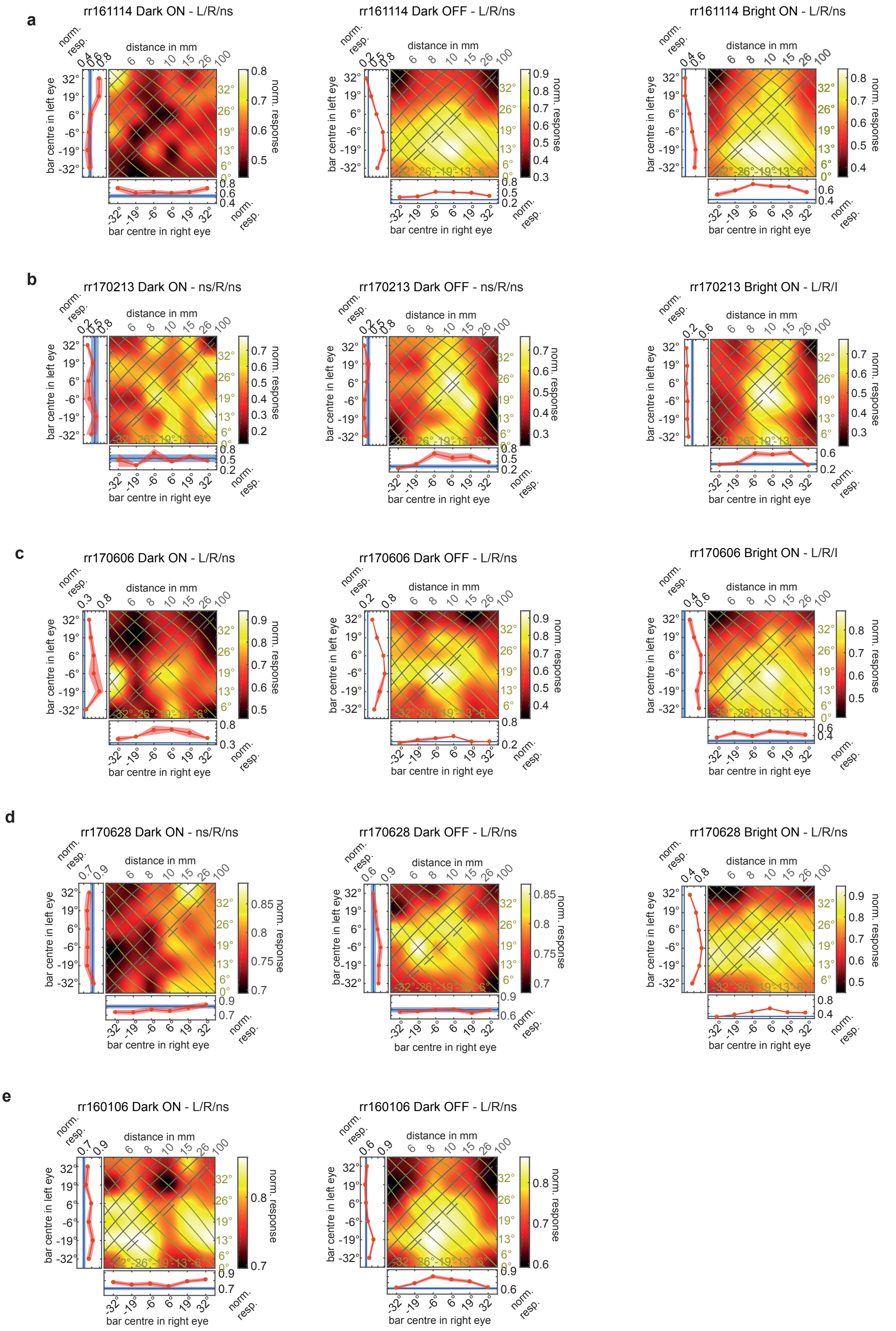

Supplementary Figure 7 | Dark bar on- and off-responses compared to bright bar on-responses in TAcen-neurons. a-e, Left panels show monocular and binocular response field plots for TAcen-neurons that have been tested for dark bar responses. Response field headers state neuron ID, stimulus type and outcome of two-way-ANOVA with “L” (“R”) being significant left (right) eye input and “I” significant interaction term (see Table 1), otherwise “ns” meaning not significant. Left panels show dark bar on-responses (normalized spike count in 250ms time window starting at 1ms after stimulus onset), middle panels show dark bar off-responses (spike count in 200ms time window starting 51ms after dark bar vanished). **a-d,** Right panels show bright bar on-responses (normalized spike count in 250ms time window starting at 1ms after stimulus onset).

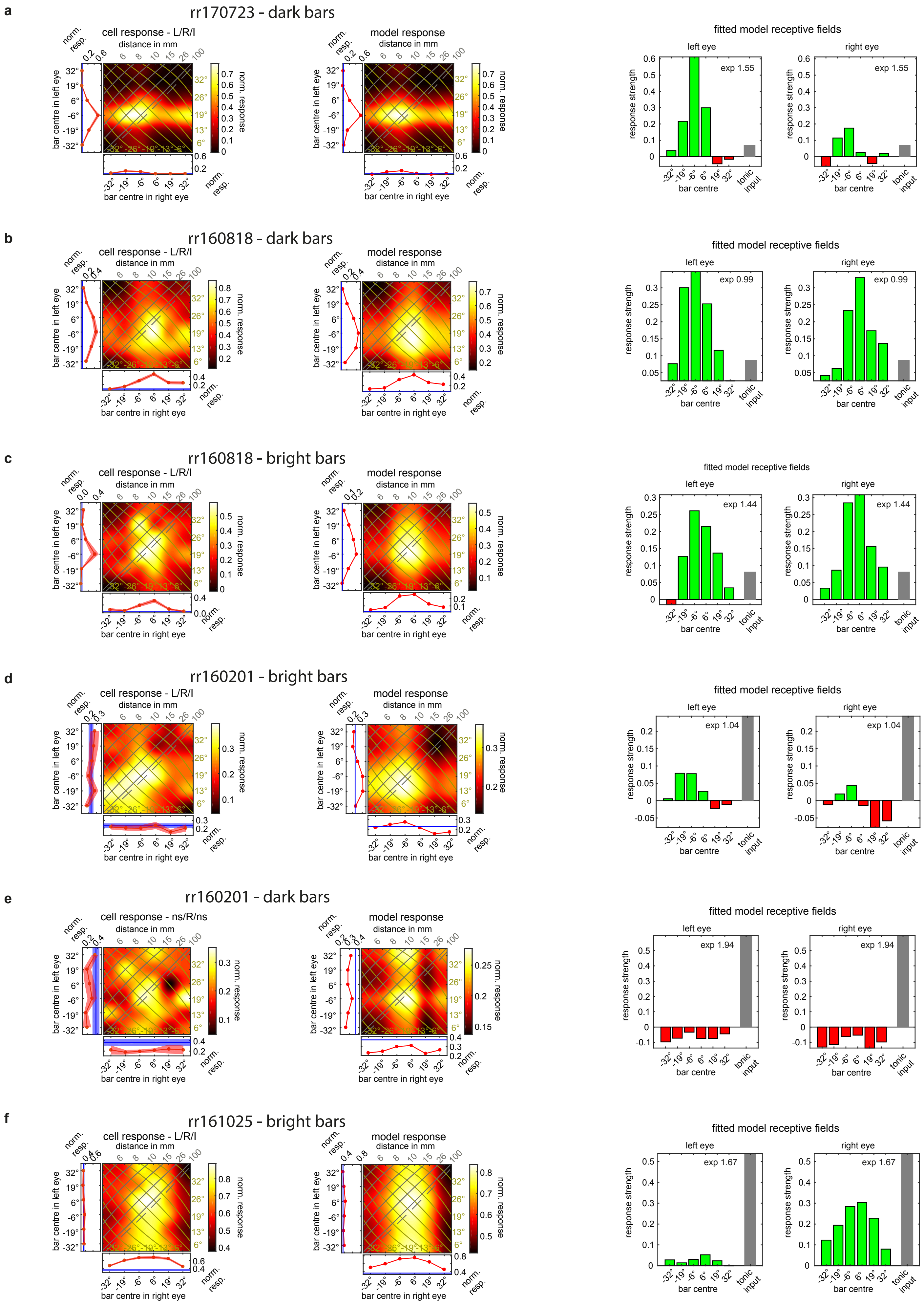
a
rr170723 - dark bars
cell response - L/R/I
model response
distance in mm
norm. resp.
norm. response
bar centre in left eye
bar centre in right eye
fitted model receptive fields
left eye
right eye
exp 1.55
response strength
bar centre
tonic input
b
rr160818 - dark bars
cell response - L/R/I
model response
fitted model receptive fields
exp 0.99
c
rr160818 - bright bars
cell response - L/R/I
model response
fitted model receptive fields
exp 1.44
d
rr160201 - bright bars
cell response - L/R/I
model response
fitted model receptive fields
exp 1.04
e
rr160201 - dark bars
cell response - ns/R/ns
model response
fitted model receptive fields
exp 1.94
f
rr161025 - bright bars
cell response - L/R/I
model response
fitted model receptive fields
exp 1.67

Supplementary Figure 8 | Response field plots and fitted receptive fields for TMEcen-neurons. a-f, Left panels show monocular and binocular response field plots for four TMEcen-neurons. Response field headers state neuron ID and outcome of two-way-ANOVA with “L” (“R”) being significant left (right) eye input and “I” significant interaction term (see Table 1), otherwise “ns” meaning not significant. Middle panels show model predictions and right panels fitted receptive fields for left and right eye with excitations (green bars) and inhibitions (red bars) at corresponding azimuthal locations (x-axis). Grey bars show tonic input (shown in both RF plots but applied only once). Exponent in upper right corner (exp). Negative (positive) values on x-axis indicate locations left (right) of centre.

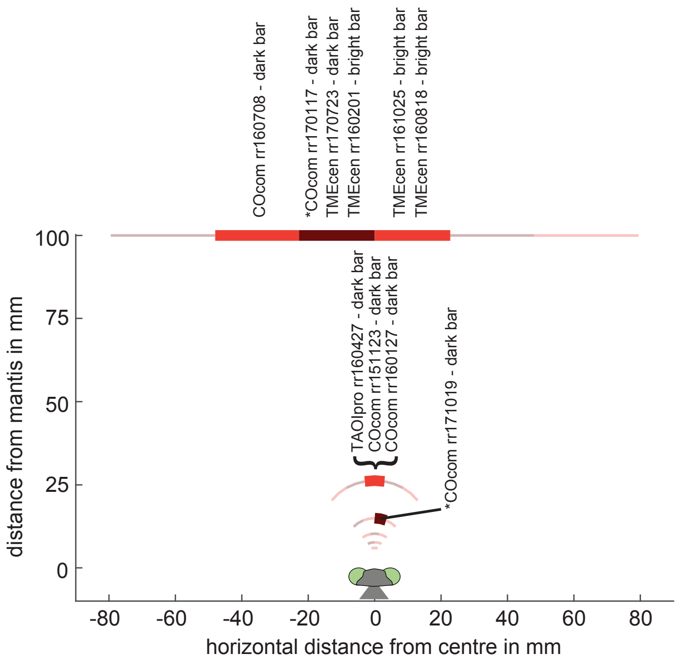

Supplementary Figure 9 | Peak responses of mantis neurons to simulated bar location. All simulated bar locations are shown in top view as presented in Fig. 1f. Bold bars represent those which elicited the strongest response in the raw (noninterpolated) binocular response fields of those neurons that are indicated by type and ID. *for tuned inhibitory neurons COcom rr170117 and rr171019 the simulated bar location with the deepest dip in the “stripe” of the binocular response field is plotted.

Supplementary references

1. Cardona, A. *et al.* An integrated micro-and macroarchitectural analysis of the Drosophila brain by computer-assisted serial section electron microscopy. *PLoS Biol.* **8,** e1000502 (2010).
