# Supplementary material for: A neuronal correlate of insect stereopsis
Source: Nat Commun. 2019 Jun 28;10:2845. doi: 10.1038/s41467-019-10721-z (PMC6599392; doi:10.1038/s41467-019-10721-z)
Supplement: Supplementary file 2 — Reporting Summary [file 41467_2019_10721_MOESM2_ESM.pdf]

Corresponding author(s): Ronny Rosner

Last updated by author(s): May 28, 2019

Reporting Summary

Nature Research wishes to improve the reproducibility of the work that we publish. This form provides structure for consistency and transparency in reporting. For further information on Nature Research policies, see Authors & Referees and the Editorial Policy Checklist.

Statistics

For all statistical analyses, confirm that the following items are present in the figure legend, table legend, main text, or Methods section.

n/a	Confirmed	
☐	☒	The exact sample size (n) for each experimental group/condition, given as a discrete number and unit of measurement
☐	☒	A statement on whether measurements were taken from distinct samples or whether the same sample was measured repeatedly
☐	☒	The statistical test(s) used AND whether they are one- or two-sided<br>*Only common tests should be described solely by name; describe more complex techniques in the Methods section.*
☐	☒	A description of all covariates tested
☐	☒	A description of any assumptions or corrections, such as tests of normality and adjustment for multiple comparisons
☐	☒	A full description of the statistical parameters including central tendency (e.g. means) or other basic estimates (e.g. regression coefficient) AND variation (e.g. standard deviation) or associated estimates of uncertainty (e.g. confidence intervals)
☐	☒	For null hypothesis testing, the test statistic (e.g. F, t, r) with confidence intervals, effect sizes, degrees of freedom and P value noted<br>*Give P values as exact values whenever suitable.*
☒	☐	For Bayesian analysis, information on the choice of priors and Markov chain Monte Carlo settings
☒	☐	For hierarchical and complex designs, identification of the appropriate level for tests and full reporting of outcomes
☒	☐	Estimates of effect sizes (e.g. Cohen's d, Pearson's r), indicating how they were calculated

Our web collection on statistics for biologists contains articles on many of the points above.

Software and code

Policy information about availability of computer code

Data collection	Neuronal recordings were done with Spike 2 versions 7.12 - 7.20 (Cambridge Electronic Design).
Data analysis	Neuronal recordings were analysed with Spike 2 versions 7.12 - 7.20 (Cambridge Electronic Design) and custom written code in Matlab 2017b (MathWorks). Code for evaluating the also provided data and for generating figures is available under DOI: 341 10.25405/data.ncl.8063327. Microscopy data was analysed with Amira 5.33 (Thermo Fisher Scientific).

For manuscripts utilizing custom algorithms or software that are central to the research but not yet described in published literature, software must be made available to editors/reviewers. We strongly encourage code deposition in a community repository (e.g. GitHub). See the Nature Research guidelines for submitting code & software for further information.

Data

Policy information about availability of data

All manuscripts must include a data availability statement. This statement should provide the following information, where applicable:

- Accession codes, unique identifiers, or web links for publicly available datasets
- A list of figures that have associated raw data
- A description of any restrictions on data availability

Reconstructed neurons are available through NeuroMorpho.org under DOI: 10.13021/ay7p337fw49 and neurophysiological data under DOI: 10.25405/data.ncl.8063327.

Field-specific reporting

Please select the one below that is the best fit for your research. If you are not sure, read the appropriate sections before making your selection.

☒ Life sciences ☐ Behavioural & social sciences ☐ Ecological, evolutionary & environmental sciences

For a reference copy of the document with all sections, see nature.com/documents/nr-reporting-summary-flat.pdf

Life sciences study design

All studies must disclose on these points even when the disclosure is negative.

Sample size	The number of neurons is typical for intracellular studies with neuronal stainings and reconstructions. The number of repetitions per neuron achieved the statistical power necessary to demonstrate disparity tuning (our key finding).
Data exclusions	All recordings with unequivocal neuronal stainings of the described types of neuron were included in the study.
Replication	All recordings were done in different animals. For each cell we collected responses to at least 10 repetitions of the the main test condition (dark or bright bars) for that particular cell.
Randomization	Bar stimuli were organized in pseudorandom order. The spiralling disc was shown in alternating order (control condition, near condition, control condition, near condition, ...) to prevent possible neuronal activity drift to influence the results.
Blinding	Visual stimuli were provided automatically in pseudorandom order (bar stimuli) or as alternating sequence (spiralling disc). Stainings were done after the recordings had finished. Thus, the neuron identity was not known to the experimenter during run time of the recording.

Reporting for specific materials, systems and methods

We require information from authors about some types of materials, experimental systems and methods used in many studies. Here, indicate whether each material, system or method listed is relevant to your study. If you are not sure if a list item applies to your research, read the appropriate section before selecting a response.

Materials & experimental systems

n/a	Involved in the study	
☒	☐	Antibodies
☒	☐	Eukaryotic cell lines
☒	☐	Palaeontology
☐	☒	Animals and other organisms
☒	☐	Human research participants
☒	☐	Clinical data

Methods

n/a	Involved in the study	
☒	☐	ChIP-seq
☒	☐	Flow cytometry
☒	☐	MRI-based neuroimaging

Animals and other organisms

Policy information about studies involving animals; ARRIVE guidelines recommended for reporting animal research

Laboratory animals	The study was done with adult praying mantids (female Hierodula membranacea; female and male Rhombodera megaera).
Wild animals	The study did not involve wild animals.
Field-collected samples	The study did not involve samples collected from the field.
Ethics oversight	No ethics approval was required because the research was carried out on insects.

Note that full information on the approval of the study protocol must also be provided in the manuscript.
